# Feasibility of continuous glucose monitoring in children with diabetic ketoacidosis: an exploratory observational study

**DOI:** 10.1007/s00431-025-06368-2

**Published:** 2025-08-15

**Authors:** Verónica Izquierdo, Nerea Afonso-Bouza, Eva María Montoto-Méndez, Graciela Gómez-Silva, Marcos Pazos-Couselo, Antonio Rodríguez-Nuñez

**Affiliations:** 1 Simulation, Life Support and Intensive Care Research Unit of Santiago de Compostela (SICRUS), Health Research Institute of Santiago de Compostela (IDIS), Departamento de Psiquiatría, Radiología, Salud Pública, Enfermería y Medicina, Universidade de Santigo de Compostela, Santiago de Compostela, Spain; 2 Faculty of Nursing, Department of Psychiatry, Radiology, Public Health, Nursing and Medicine, University of Santiago de Compostela, Av/Xoan XXIII, s/n. 15782, Santiago de Compostela, Spain; 3 Spanish Network in Maternal, Neonatal, Child and Developmental Health Research (RICORS-SAMID, RD24/0013/0023), Instituto de Salud Carlos III, Madrid, Spain; 4 Pediatric Critical, Intermediate and Palliative Care Section, University Hospital of Santiago de Compostela, SERGAS, Santiago de Compostela, Spain; 5 CLINURSID Research Group, University of Santiago de Compostela, Santiago de Compostela, Spain

**Keywords:** Diabetic ketoacidosis, Children, Critical Care, Continuous glucose monitoring, Type I diabetes, New-onset

## Abstract

Diabetic ketoacidosis (DKA) is a life-threatening complication of diabetes and a leading cause of Pediatric Intensive Care Unit (PICU) admissions. The use of continuous glucose monitoring (CGM) during the acute and critical phase of DKA has been rarely explored and remains uncertain due to concerns about accuracy and utility in a setting where frequent capillary glucose measurements are standard practice. Data was collected from medical records of patients admitted to the PICU with new-onset DKA as the initial presentation of type 1 diabetes (T1D). Mean absolute relative difference (MARD) and Clarke Error Grid (CEG) analysis were used to assess CGM accuracy. Data from 19 patients (mean age 9.9 ± 3.4 years) were included. Within the first 48 h, 16 hypoglycemic episodes were recorded, with CGM detecting 14 episodes and capillary glucose detecting two. A total of 238 matched pairs of capillary and CGM interstitial glucose values were analyzed. Statistical analysis found capillary glucose values significantly higher than interstitial values (*p* < 0.001). The overall MARD was 14.5% and CEG analysis indicated 89.1% of matched pairs within zones A and B.

* Conclusions*: CGM might be a useful point-of-care tool that provides valuable information that may help clinicians to make timely management decisions. The ability of CGM to indicate trends in glucose fluctuations could be its main clinical advantage, particularly in anticipating and preventing potentially dangerous hypoglycemic events, thereby optimizing patient management and safety.

**Table:** 

**What is Known:**
• *DKA emergencies require close glucose monitoring. Standard methods, such as capillary glucose monitoring or venous blood glucose measurements, have some limitations in terms of comfort, frequency, and trend detection.* • *CGM is currently rarely used in PICU or DKA due to a lack of clinical trials, resulting in uncertainty about its accuracy in pediatric DKA. Additionally, CGM has not been FDA-approved for use in inpatients and to manage diabetes emergencies.*
**What is New:**
• *CGM may benefit children with DKA from the onset.* • *DKA management in PICUs by showing glucose trends and enabling hypoglycemia to be detected early, supporting timely interventions, reducing workload, and minimizing patient discomfort through fewer capillary punctures.*

• *DKA emergencies require close glucose monitoring. Standard methods, such as capillary glucose monitoring or venous blood glucose measurements, have some limitations in terms of comfort, frequency, and trend detection.*

• *CGM is currently rarely used in PICU or DKA due to a lack of clinical trials, resulting in uncertainty about its accuracy in pediatric DKA. Additionally, CGM has not been FDA-approved for use in inpatients and to manage diabetes emergencies.*

• *CGM may benefit children with DKA from the onset.*

• *DKA management in PICUs by showing glucose trends and enabling hypoglycemia to be detected early, supporting timely interventions, reducing workload, and minimizing patient discomfort through fewer capillary punctures.*

## Introduction

Diabetic ketoacidosis (DKA) is a potentially life-threatening complication of diabetes, commonly occurring at the time of type 1 diabetes (T1D) diagnosis and representing the leading cause of endocrine admission to the Pediatric Intensive Care Unit (PICU) [1, 2]. DKA management requires meticulous clinical and metabolic monitoring, as well as individualized fluid replacement and insulin infusion to gradually restore metabolic stability while minimizing the risk of neurological complications [2].

Intermittent repetitive capillary glucose measurement has traditionally been the standard method for glucose monitoring in PICUs [3–7] but must be crosschecked against venous blood glucose concentrations [2]. Although continuous glucose monitoring (CGM) is the standard method of glucose monitoring in the outpatient setting [8], it has not yet been widely implemented for inpatient care in critically ill children.

CGM offers practical advantages, including non-invasiveness and the ability to provide clinically meaningful information about continuous glucose levels and, perhaps more importantly, glucose trends, with trend arrows indicating both direction and velocity of glucose fluctuations [9, 10]. While CGM has been explored in adult Intensive Care Unit (ICU) patients [7, 11–17], its use in critically ill children and adolescents, particularly those presenting with DKA, remains limited.

A limited number of retrospective [5, 8, 18] and prospective [10, 19] studies have evaluated CGM feasibility and accuracy in pediatric inpatient settings during DKA admissions. However, data on its implementation in newly diagnosed T1D patients requiring PICU admission remain scarce.

With the hypothesis that CGM implementation would be both feasible and informative for the PICU healthcare team, we collected data from a cohort of children with DKA admitted to our PICU. Our primary objective was to assess the feasibility, usefulness, and accuracy of CGM in the initial management of DKA.

## Methods

### Study design and patients

A retrospective observational descriptive study was conducted to collect data from the medical records of pediatric patients (aged < 16 years) who were admitted to our PICU with new-onset DKA as the initial presentation of T1D, between January 2021 and December 2024 at the University Clinical Hospital of Santiago de Compostela, Spain. Children with diabetes who presented with DKA due to an intercurrent illness or inadequate outpatient management were excluded.

All patients were treated in accordance with the institutional DKA management protocol in the PICU that includes point-of-care (POC) capillary blood glucose values, obtained via the Accu-Chek® Performa meter (Roche Diagnostics, Basel, Switzerland) and from 2021 the placement of a commercial CGM at the time of PICU admission.

The study was approved by the Ethics Committee for Research with Medicinal Products of Galicia (CEIm-G) (reference number 2024/321) and conducted in accordance with the Declaration of Helsinki and Good Clinical Practice. Informed consent from parents and assent by patients was not required for this study, as the data were collected through a retrospective chart review, with no personally identifiable information included in the analysis.

This article follows the STROBE guidelines for the reporting of observational studies.

### Study devices and procedures

We used a FreeStyle Libre® 2 (Abbott Laboratories, IL, USA), a factory calibrated intermittently scanned CGM device intended to last for 14 days, which was applied to the patients’ upper arm within two hours of admission to the PICU. This device comprises a sensor with an integrated transmitter and receiver. After placement, the device requires a 1-h warm-up and a stabilization period. Afterwards, the PICU nurses used handheld readers to start the sensor and register the interstitial glucose data.

Predictive and indicative alarms were configured for hypoglycemia and hyperglycemia, with thresholds set at 80 mg/dL or hypoglycemia and 200 mg/dL for hyperglycemia.

Freestyle Libre® 2 is approved for use in children aged ≥ 2 years. In this study, two patients under 2 years of age received the device following individualized assessment conducted jointly by a pediatric intensivist and a pediatric endocrinologist, with compassionate off-label use through shared decision-making with the parents, as is permitted by legislation. Prior studies support the feasibility and potential clinical value of CGM in infants outside the approved age range [20, 21].

DKA was defined as hyperglycemia (blood glucose > 200 mg/dL), elevated ketonemia (> 3 mmol/L), and metabolic acidosis with increased anion gap: venous pH < 7.3 and/or bicarbonate (HCO_3_) < 15 mEq/L.

### Statistical analysis

Descriptive data are presented as mean and standard deviation (SD) for continuous variables and as counts (%) for categorical variables. To test differences between pairs of POC capillary and CGM interstitial glucose values, we used a Wilcoxon signed-rank test.

Accuracy was assessed using the mean absolute relative difference (MARD) and the Clarke Error Grid (CEG) analysis. MARD was calculated as the mean absolute difference between CGM and reference glucose values, with smaller values reflecting better precision [5, 22]. CEG was used to quantify the clinical accuracy of blood glucose measurements compared to a reference glucose value. A common application of CEG is the calculation of the percentage of values falling within zones A and B. Zone A includes values within 20% of the reference glucose level. Zone B includes values outside of 20%, but do not result in inappropriate treatment [18, 23, 24].

POC glucose values and CGM values were paired to calculate the MARD and the CEG. The POC capillary glucose value was included in the analysis if a corresponding CGM interstitial measurement was recorded within a ± 5-min window of the POC glucose reading.

We calculated the overall MARD between POC capillary blood glucose and CGM interstitial glucose values. MARD values were compared across 12-h intervals during the PICU admission. Differences between intervals were assessed using the Friedman test. Statistical analyses were performed using IBM SPSS Statistics for Windows (version 29.0.1.0; IBM Corp., Armonk, NY) and R Studio version R 4.4.2.

## Results

Nineteen children were admitted to the PICU for DKA as the initial presentation of T1D during the study period. Their mean age was 9.9 years (range: 8 months to 14 years) and 9 (47%) were male. The mean HbA1c and mean capillary glucose upon admission were 12.4% ± 2.9 and 479.7 ± 131.0 mg/dL respectively. DKA was severe in 8 cases, moderate in 8 and mild in 3 patients. Additional demographic and clinical characteristics are summarized in Table 1.

**Table 1:** Patient baseline characteristics (*N* = 19)

Characteristic	Mean + SD, or *n* (%)
Age, years	9.9 ± 3.4
Gender, male, *n* (%)	9 (47%)
Weight (kg)	30.5 ± 16.4
Height (cm)	129.8 ± 28.6
BMI (kg/m^2^)	16.5 ± 3.1
Weight Z-score	− 0.46 ± 1.33
Admission laboratory data	
HbA1c (%)	12.4 ± 2.8
Glucose (mg/dL)	479.7 ± 131.0
pH	7.14 ± 0.13
Bicarbonate (mmol/L)	8.2 ± 4.9
Lactate (mmol/L)	2.1 ± 0.99
Ketone (mmol/L)	5.9 ± 1.0
Total POC capillary blood glucose measurements (*n*)*	15.48 ± 9.3
Total CGM measurements (*n*)	32.0 ± 19.0
Total serum measurements (*n*)	2.4 ± 1.5

*Performed on a blood gas analyzer. Data are presented as mean ± SD or number of patients (%). *BMI*, body mass index; *HbA1c*, hemoglobin A1c; *SD*, standard deviation

The length of stay in the PICU was 39.3 ± 21.2 h and the overall hospital length of stay was 7.2 ± 2.1 days. No system malfunction, incidents, or related complications, and no mortality or adverse effects were observed during the PICU stay. When children were discharged from the PICU to the ward, the CGM was maintained, and management was led by the pediatric endocrinology team.

The isCGM sensor (FreeStyle Libre® 2) was applied within 2 h of admission to the PICU. Due to the required warm-up period, CGM glycemic data were considered starting from the second hour after sensor placement. CGM data labeled as “High” (> 500 mg/dL) were excluded from the analysis, and no “Low” (< 40 mg/dL) values were reported during the study period. These labels replace numeric values when readings fall outside the sensor’s measurable range. The mean total measurements, including POC capillary blood glucose concentration, blood gas analysis, interstitial, and biochemical glucose values are presented in Table 1.

**Fig. 1:**
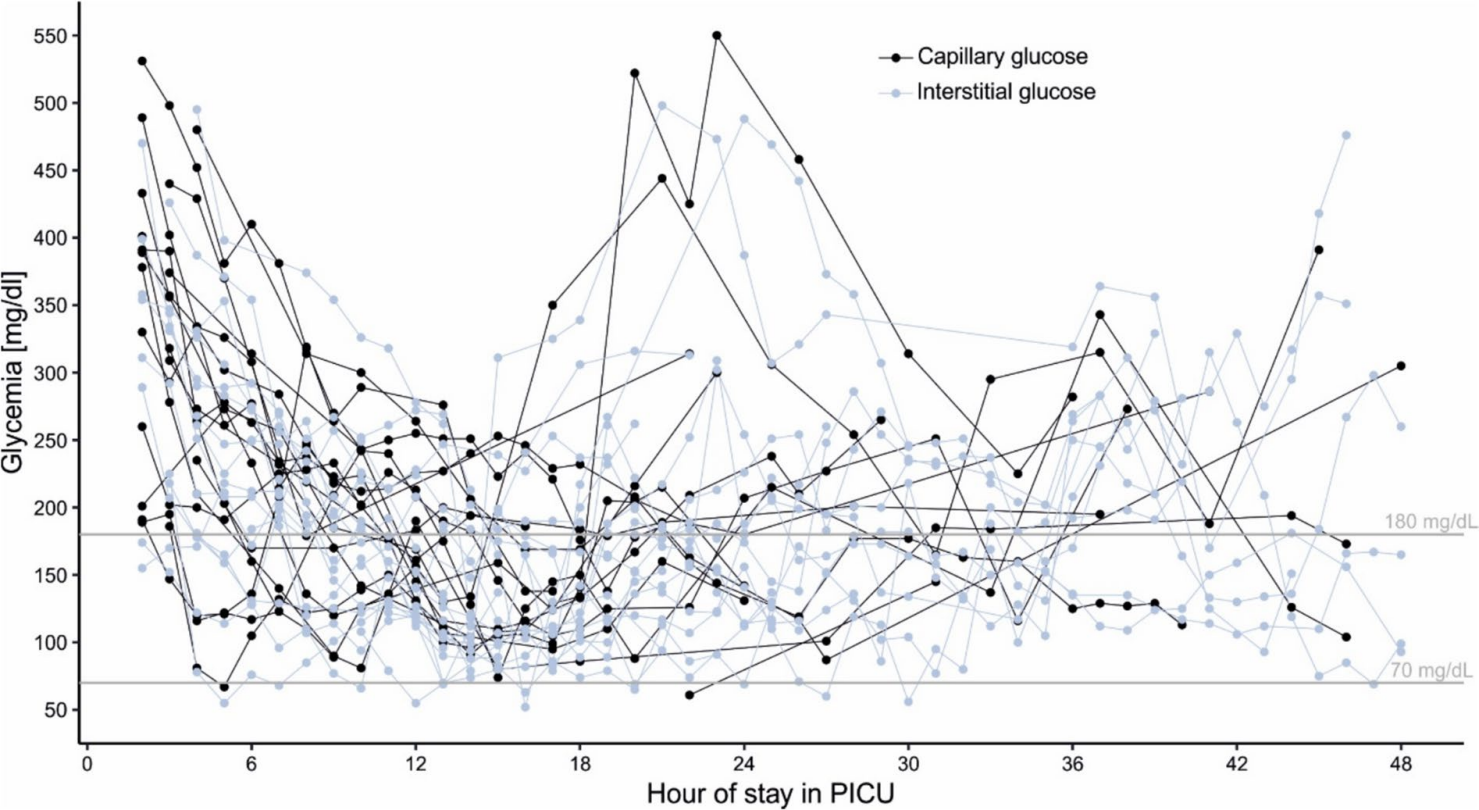
Total capillary and interstitial glucose values within the first 48 h of PICU admission (*N* = 19)

A total of 899 POC capillary and CGM measurements were performed during the study. During the first 48 h of PICU admission, 795 glucose measurements were performed, including 275 POC capillary measurements and 520 CGM interstitial measurements. Figure 1 presents the capillary and interstitial glucose values for the nineteen patients during this period.

The frequency of capillary fingerpricks decreased over time, while the measurements of interstitial glucose maintained or increased during the stay of patients in the PICU.

Sixteen episodes of hypoglycemia (≤ 70 mg/dL) were recorded within the first 48 h, 14 episodes with CGM (87.5%), and two with POC capillary glucose. In one instance, both CGM and capillary glucose measurement coincided in registering hypoglycemia in the same patient within the same hour. In another case, hypoglycemia was recorded exclusively by capillary glucose measurement but was not with CGM (Fig. 1). Clinical manifestations of hypoglycemia, such as dizziness, sweating, or tremors, were systematically assessed in each case but were not observed or reported during any episode.

**Fig. 2:**
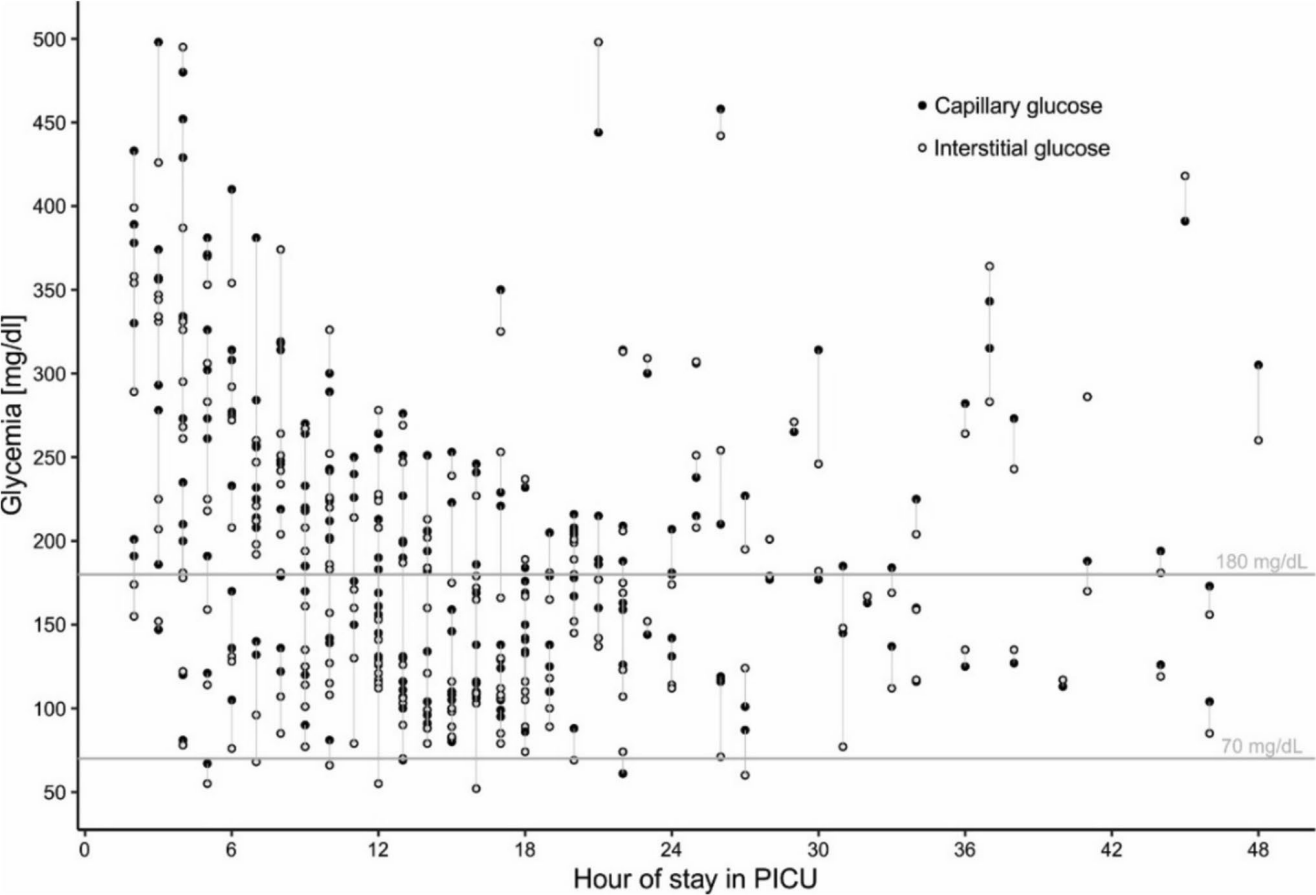
Paired capillary and interstitial glucose values during the first 48 h of PICU admission (*N* = 19)

A total of 238 paired POC capillary and CGM interstitial glucose values, matched within a ± 5-min window, were analyzed. Figure 2 shows the paired glucose values within the first 48 h of PICU admission. Statistical analysis demonstrated a significant difference between capillary and interstitial glucose values (*p* < 0.001, Wilcoxon test). Capillary glucose values (206.2 ± 88.5) were higher than interstitial glucose values (185.1 ± 89.8).

An analysis of capillary glucose values ≤ 100 mg/dL (14 events in 7 patients) also showed significantly higher capillary values (85.9 ± 11.3 mg/dL) compared to corresponding interstitial glucose values (74.9 ± 11.9 mg/dL) (*p* < 0.016, *N* = 14, Wilcoxon test).

As expected, glycemic values decreased as patients received intensive care and monitoring in the PICU, including intravenous insulin infusion, fluid replacement, electrolyte correction, and continuous glucose monitoring (Fig. 2).

The overall mean MARD was 14.5% ± 12.7%. MARD values were analyzed across 12-h intervals during the PICU admission period, showing a progressive decrease over time (Table 2). Although no statistically significant differences were observed across time intervals (*p* = 0.06, Friedman test), the trend approached statistical significance.

**Table 2:** MARD values by time interval during PICU admission (*N* = 238)

Time interval since admission	MARD (%) + SD
2–12 h	15.5 ± 12.7
13–25 h	14.7 ± 13.3
26–39 h	12.8 ± 12.3
≥ 40 h	10.5 ± 9.8

*MARD*, mean absolute relative difference; *SD*, standard deviation

The CEG analysis of the 238 time-matched POC capillary glucose and CGM revealed 89.1% of readings were within zone A and zone B. This means clinically accurate values leading to correct treatment decisions.

## Discussion

DKA is a life-threatening hyperglycemic emergency that requires rapid and appropriate treatment. While the fundamental approach to managing DKA has remained largely unchanged for decades, the method for glucose monitoring remains a topic of debate [6].

In DKA, the traditional recommended monitoring methods are capillary blood glucose measurements and venous blood samples, taken hourly during the initial hours and every 2 h once glucose levels approach the normal range and DKA begins to resolve [2, 9, 25]. However, frequent fingersticks cause children’s discomfort and increased nursing workload [5], which has been associated with increased errors and decreased patient safety [12]. Our findings indicate that the nurse team progressively reduced the frequency of capillary glucose measurements as patients stabilized and acidosis resolved. Although not a primary objective, this potential reduction in nurses’ workload in the PICU could be relevant.

A potential advantage of CGM is its ability to show down-trending values and rapid glucose excursions in real time, which may help clinicians intervene earlier, thereby reducing the frequency of hypoglycemic episodes and minimizing the risk of neurological complications. Additionally, CGM may be useful in confirming that blood glucose levels are decreasing in response to treatment and can indicate when PICU staff should perform capillary or venous blood glucose measurement due to the possibility of impending hypoglycemia.

In our study, a total of 795 glucose measurements were obtained via capillary and CGM during the first 48 h of admission. Of these, 16 episodes of hypoglycemia (≤ 70 mg/dL) were recorded (14 with CGM, 2 with POC capillary glucose, and just one agreement between both methods). These apparent “false positives” may serve as precautionary alerts that could enhance patient safety, prompting timely standard glucose verification.

Additionally, an analysis of 238 matched pairs of capillary and interstitial glucose values throughout the PICU stay revealed a statistically significant difference (*p* < 0.001), with capillary glucose higher than interstitial values. This absolute difference of the mean values of approximately 21 mg/dL, though statistically significant, falls within a range unlikely to influence clinical decision-making, particularly in the context of DKA management where continuous monitoring and trends may provide more comprehensive information than isolated values.

This discrepancy must be acknowledged by practitioners and highlights the relevance of confirming hypoglycemia through additional commonly used methods, such as venous blood samples, while also assessing clinical symptoms like sweating, tremors, or dizziness. CGM is a tool that provides a greater amount of data, including predictive trend alerts, allowing for earlier detection and intervention. This acknowledgement of the strengths and limitations of each method, and the proactive actions, allows the PICU team to intervene on time, enhancing patient safety.

On the other hand, it is important to remember that CGM devices measure glucose in interstitial fluid, which can result in some lag time, especially during periods of rapid glucose fluctuations common in critically ill patients [18] and initial phase of T1D, as in our study sample.

Regarding the relevant concern about CGM accuracy during DKA, our study found an overall MARD of 14.5% when comparing the FreeStyle Libre 2 and POC capillary glucose measurements. Recent studies in pediatric patients admitted to a PICU have reported MARD values of 7.9% [8], 11.8% [18], 13.0% [5], and 20% [19]. However, these studies involved different patient profiles, clinical conditions, and CGM devices. In critical care settings, including PICU, even during periods of highest acidosis and increased glucose variability, a MARD of 13% is often considered clinically acceptable and valid for inpatient management and comparable to previously published adult data [18].

In our study, we compared MARD values across 12-h intervals during PICU admission and found no statistically significant differences (*p* = 0.06). However, the first 12 h had the highest MARD, which progressively declined. This period coincides with the initiation of intravenous (IV) insulin and fluid administration, glucose fluctuations, and impaired perfusion, factors that may affect CGM accuracy [18]. A recent study also reported higher MARD values during IV insulin administration [18]. Given this, PICU protocols should not solely rely on CGM for glucose management during acute phase admission. Conversely, as clinical stability improves and MARD values decrease, CGM may be more reliable for guiding glucose management later in the PICU course.

Additionally, our results indicate that 89.1% of matched pairs were within the clinically acceptable A and B zones of the Clarke Error Grid. This is a figure lower than other prior studies in PICU that reported CEG values of 95% [10], 97.6% [18], 98% [8], and 98.5% [5].

A study of Parkes et al. communicated that in an adult ICU, a MARD below 15% is considered accurate, although a consensus is still needed [26]. Evidence in the adult ICU population using CGM has demonstrated positive CGM accuracy [17, 27, 28]. Paradoxically, the use of CGM in children has been anecdotal and the few available data refer to its accuracy and not to its practical usefulness in the PICU [5, 29, 30].

The novelty of our findings also lies in the exclusive inclusion of patients with newly diagnosed T1D. Despite being a preliminary clinical observational study, our findings align with previous research that both CGM and POC capillary glucose could provide valuable information for treatment decisions and that CGM should facilitate earlier intervention. Our findings suggest that while CGM may not completely replace capillary glucose monitoring in the acute phase of DKA management, it may provide clinically valuable information that can help to optimize just-in-time treatment adjustments, prevent both hypoglycemia and hyperglycemia events, potentially improve patient care and outcomes, and reduce the burden of PICU nursing staff. Adding its relative ease of use in the hospital setting and considering the highly equipped ICU environment, we agree with Patham et al. that CGM glucose values could be proposed as a fifth vital sign [9] in a pediatric ICU setting as well.

Our study has limitations that should be considered when interpreting the results. First, this is a preliminary clinical observational study, not designed or powered to support definitive conclusions, especially regarding the technical aspects of the CGM sensors. The small sample size (*n* = 19 with 238 POC capillary glucose and CGM paired measurements) and the single-center limit the generalizability of our findings. Additionally, we did not have data on sensor active time. Another limitation is that capillary glucose measurements were used as the reference method instead of a reference blood glucose analyzer. This highlights the need for larger and multicenter studies, including CGM glucometrics, interventions prompted directly by CGM data, and randomized clinical trials.

## Conclusions

Newly diagnosed pediatric patients with DKA may benefit from minimally invasive glucose monitoring from the outset. Although CGM glucose values differed from corresponding capillary measurements, CGM demonstrated acceptable accuracy. Its ability to indicate glycemic excursions and detect rapid declines in glucose levels could be its main clinical advantage, linked to anticipation and treatment of hypoglycemia events. However, this requires healthcare personnel to be appropriately trained with its operational methods in order to interpret the results accurately.

## Data Availability

No datasets were generated or analysed during the current study.

## Abbreviations

CEG: Clarke Error Grid
CGM: Continuous glucose monitoring
DKA: Diabetic ketoacidosis
MARD: Mean absolute relative difference
PICU: Pediatric Intensive Care Unit
POC: Point-of-care
T1D: Type 1 diabetes
